# How does strategic flexibility affect bricolage: The moderating role of environmental turbulence

**DOI:** 10.1371/journal.pone.0238030

**Published:** 2020-08-28

**Authors:** Mengmeng Meng, Jiasu Lei, Jie Jiao, Qiuyan Tao

**Affiliations:** 1 School of Network Education, Beijing University of Posts and Telecommunications, Beijing, China; 2 School of Economics and Management, Tsinghua University, Beijing, China; 3 School of Management, Beijing Union University, Beijing, China; Shandong University of Science and Technology, CHINA

## Abstract

While most studies have viewed strategic flexibility as a capability to cope with the environmental turbulence and promote the product innovation, few of them investigate the mediating mechanism in the relationship between the strategic flexibility and product innovation. According to the resource-based view, we regard the bricolage as a concrete activity of recombining the different resources in the product development process and explore the underlying mechanism. Our results reveal that strategic flexibility has a positive and significant effect on bricolage. The effect of strategic flexibility on product innovation is partially mediated by bricolage. Furthermore, environmental turbulence moderates the relationship between strategic flexibility and bricolage positively. We contribute to the strategic flexibility research by exploring the effect of strategic flexibility on the bricolage and product innovation form a mediating perspective and offering a more nuanced and in-depth understanding of the impact of strategic flexibility. This research also provides new evidence on the effect of strategic flexibility on product innovation in transition economies such as China, where strategic flexibility is essential for firms to adapt to an uncertain environment.

## 1. Introduction

How does strategic flexibility matter in the product competition? According to Sanchez's (1995) concept of strategic flexibility, the flexibilities associated with product creation resources and enterprise's capability to coordinate these resources play a fundamental role in product competition. In essence, strategic flexibility empowers firms to respond quickly to changing competitive conditions by reconfiguring the resources chain and utilizing the opportunities in a turbulent environment [1, 2]. In particular, strategic flexibility gives rise to distinct innovation [3] and firm performance [4]. For example, flexible firms tend to expand international product networks [5] and perform better in product innovation [6, 7]. Besides, Yawson and Greiman (2017) regard the strategic flexibility as a scenario analysis tool and describe its use in human resources development and management [8]. Therefore, extensive studies view strategic flexibility as a significant organizational capability to achieve competitive advantage [9]. Especially in a transition economy, where the environment is characterized by volatility and uncertainty, strategic flexibility is a more feasible capability and option to cope with the resource constraint triggered by technological change and demand variation.

Previous studies have provided insight into various outcomes of strategic flexibility and various outcomes that mainly focus on firm performance [10], new product development [11], and radical innovation [3] in the rapidly changing environment. These studies offer empirical evidence regarding strategic flexibility as an antecedent to the innovation [3, 7]. Strategic flexibility, as a combinative capability, helps firms well redeploy resources and reconfigure existing operation routines, which increases the new product introduction [12, 13] and reduce the reaction time in responding to the complicated dynamic environment. Nevertheless, previous studies overlook the underlying mechanisms through which strategic flexibility affects product innovation. The literatures have focused exclusively on the ultimate output performance, such as financial performance [1], competitive advantage [9, 10], ignoring the intermediate process. Strategic flexibility must work together with organizational capabilities to affect product innovation indirectly [6]. So it's necessary to reveal the intermediate factors that channel the effect of strategic flexibility on product innovation. However, it surprisingly receives less attention in the existing studies.

To address this deficiency, we investigate the underlying mechanism that explains how strategic flexibility matters from the perspective of resource utilization. We posit a novel channel through which strategic flexibility affects product innovation and introduces the bricolage as a mediator. According to Baker and Nelson's (2005) study, bricolage is defined as an entrepreneurial activity of recombining the handy resources in the product development process. Bricolage makes firm alleviate conditions of resource constraint and bring process knowledge and product knowledge with existing undervalued, slack, or discarded resources [14, 15]. Specifically, we suggest that strategic flexibility leads to bricolage through which ultimately improve the product innovation in resource constraint context and dynamic environment. From the view of the value creation process and entrepreneurial behavior in product innovation [15], we emphasize that strategic flexibility create value for bricolage accomplish challenging assignment and innovation task with the limited resources at hand.

China, with rapid growth and a dynamic and dysfunctional institutional environment, provides an ideal and suitable setting to test our hypotheses. China is undergoing the institutional transition, which refers to fundamental and widespread changes brought in the formal and informal social system that affects the firm's strategic choices [16]. The institutional transition is pervasive in emerging economies that presents a fascinating opportunity for firms in an uncertain and dynamic environment. Prior researches focus on the antecedents of strategic flexibility in emerging economies, for example, CEO personality [1], business ties to NPD alliances [17], and IT support [10]. We can, therefore, test the moderating role of environmental turbulence and identify conditions for how strategic flexibility influences the bricolage in an uncertain environment.

Overall, this research makes a significant contribution to the existing studies by theoretically and empirically, revealing the influencing mechanism linking strategic flexibility and product innovation in the turbulent environment. The majority of prior studies on the outcomes of strategic flexibility focus on the market entry [18], innovation [7, 11, 19], firm performance [2, 10, 20], energy efficiency [21] and competitive advantage [4, 9]. We complement the prior literature by exploring the new impact of strategic flexibility on the entrepreneurial behavior-bricolage. Bricolage is a significant path to product innovation, which offers a more nuanced and in-depth understanding of the impact of strategic flexibility on product innovation. We not only contribute to the literature on strategic flexibility [22] but also to the literature on related concepts such as bricolage [23] and environmental turbulence [24]. Hence, this research confirms the strategic flexibility as the antecedent of bricolage and provides new evidence regarding the bricolage as a path to innovativeness for resource-constrained firms [25]. We are identifying bricolage as a critical parameter that reveals how strategic flexibility affects product innovation and provides significant insights that broaden our understanding of bricolage. Our findings also investigate the direct effect of strategic flexibility on product innovation, which extends the study of zhou and wu (2010).

Our results also have managerial implications. Strategic flexibility affects the bricolage and product innovation positively, which helps enterprises manager gain a broader understanding of flexible resource and organizational structure in the changing circumstances. Actually, the environmental turbulence incurs both resource scarcity and new opportunity [15]. It implies that managers should creatively tackle the dynamic environment that brings in the external knowledge and various market opportunities. Indeed, strategic flexibility help firms gain benefits from environmental turbulence in term of demand variation and technical change [18].

The paper is organized as follows. Firstly, we demonstrate the theoretical background and conceptual framework. Second, the hypotheses are developed according to literature, and we illustrate the research design and methodology to that test the hypotheses, including sample and measurement. We conclude, theoretical contribution and manageable implications in the end.

## 2. Theoretical background and hypotheses development

### 2.1 Strategic flexibility

Strategic flexibility is defined as the capability to make a response to a dynamic environment through continuous changes in resource deployment and strategic actions. According to the resource-based review Strategic flexibility represents an effort to exploit valuable resources for competitive advantage [26]. It implies strategic flexibility is the firm's ex-ante capability to quickly redeploy and relocate resource and production process making the response to environmental turbulence, the threats of other entrants and technical changes [27]. So strategic flexibility highlights the flexible utilization of resources, and these resources are applied effectively in product development processes to acquire the core competitiveness in uncertain markets [6, 28]. Strategic flexibility enhances the value of the resource for innovation [3]. For instance, strategic flexibility helps Thai firms manage the economic crisis [29], and firms enter a new market in the US airline industry [18]. It is essential to develop the capabilities to satisfy the market demand, such as labor flexibility [19], supply chain flexibility [30], and manufacturing flexibility [31].

Strategic flexibility is a fundamental and essential principle to manage the uncertainty in product competition, divided into two dimensions: resource flexibility and coordination flexibility. Resource flexibility can be characterized by the scope of various products that a resource could efficiently be applied in development and manufacturing [6]. Resource flexibility is higher when the time and cost needed to switch to an alternative resource utilize lower [26, 32]. Then the coordination flexibility involves the flexibility in coordinating the deployment of product design and creation resources to adjust product strategies and thus makes to redeploy chains of resources efficiently [22].

Strategic flexibility produces a new dominant logic in product competition via market opportunity identification and dynamic efficiency in deploying resources. That means flexible firms benefit from the resource coordination processes and explore and exploit evolving opportunities confronted with the dynamic market. For example, a new information system enables SMEs to build a quick response system for strategic decisions and enhance flexibility for using market resources effectively [33, 34]. Strategic Flexibility minimizes the difficulty and cost of changing and coordinating the enterprise's resource base [35] and makes the enterprise managers apply advanced technologies in new product development (NPD) to create new market opportunities through reconfiguring and redeploying resource chains [10]. Therefore, the frequently mentioned effects of strategic flexibility are dynamic capabilities, core competence, and product innovation. Some empirical studies show that strategic flexibility, strongly contribute to innovation capabilities in the process of decision-making, such as identifying the significant resources engaged with NPD procedures, energy efficiency [21] and acquiring the new product family or grabbing the attention of consumers [13, 22]. Thereby, strategic flexibility plays an essential role in value creation and product competition.

### 2.2 Bricolage

Bricolage explains differential firm behavior and outcome under less extreme resource conditions labeled "create something from nothing" [36]. The basic assumption of bricolage is the condition of resource scarcity [15]. Bricolage refers to making do by applying combinations of handy resources to new problems and opportunities and has become increasingly prevalent in organizational theory [37, 38]. Bricolage represents a form of value creation with unrelated or underdeveloped resources during the opportunity-formation process—bricoleurs who use bricolage regard resource scarcity as both a problem and opportunity in an uncertain environment. Notably, organizational researchers have combined diverse strands of literature and methodology to improve the bricolage frames. The central theme of bricolage account for the reconfiguration and redeployment of resources for various applications than these for which they are initially intended for utilized. The recombination process of novel purposes sometimes is regarded as a significant approach driving the product innovation from existing resources [36, 39]. The studies suggest that bricolage alleviates the concern about the resource constraint through organization behaviors, including improvisation and recombining resources at hand [40].

Bricolage emphasizes employing resources in a new manner. So the Organizational capacities which help bricoleurs creatively utilize existing resource are the antecedents of bricolage, such as combinative capabilities, absorptive capacity, exploratory orientation [41], and strategic flexibility. Desa (2012) found that social firms in the context of unsupportive normative institutions are more willing to carry out bricolage to reallocate resources than these faced with supportive normative institutions [42]. When firms are confronted with resource scarcity, bricolage can result in imperfect but good-enough product solutions with external knowledge or original creative ideas, adding value to existing resources [43, 44]. The bricolage capabilities including making do with what is available at hand, improvising when reallocating various resources jointly, and collaborating with external partners, play a crucial role in the innovation process and influence the innovation outcomes, such as service innovation [14], knowledge transfer and knowledge protection [45], new-product development speed and creativity [46], opportunity identification [47], and organizational performance [48]. Bricolage may bring subjective knowledge and new perception to form new products and services, which is significant to identify entrepreneurial opportunities and satisfy new market needs. Built on the relevant theoretical construct of this paper, the hypotheses development and the overview of the conceptual framework are as follows.

### 2.3 Strategic flexibility and bricolage

We contend that strategic flexibility, the ability to redeploy and reconfigure resource, and product creation process rapidly has a subsequent effect on the firm's bricolage strategies. Strategic flexibility highlights the flexible utilization of resources and redefines product development processes [17], and that consequently increases the chance that improvisation and bricolage will occur. Resource flexibility represents less time and costs to switch over to another way of deploying resources, and a resource can be applied in various products for manufacturing and distributing [26]. Thus, entrepreneurs could effectively utilize scarce resources to employ bricolage activity in constraining environments [49].

Strategic flexibility in coordinating the mobilization of product creation resources consists of flexibilities to redefine product strategies, reconfigure chains of resources, and redeploy resources effectively. According to the RBV logic, firms access to valuable resources by engaging optimally and pursuing a satisfying result in the product creation process. Escalating resource flexibility and coordination flexibility minimizes the cost and difficulty of switching the various uses of resources, which leads to higher dynamic efficiency in reallocating available low-cost resources at hand. Therefore, enterprises with super dynamic efficiencies in recombining various resources and reconfiguring resource supply chains would prefer to engage in a higher level of bricolage.

Firms with strategic flexibility have tremendous of overlapping or redundant resources, and the RBV logic will be effective and salient in explaining an incremental level of bricolage activity. Faced with resource constraints, firms engage in bricolage by using the handy resources to provide new solutions and create a form of value creation. During the process, strategic flexibility help firms make use of both internal and external sources of resources and capabilities [50] and thereby increased the effect of value creation via bricolage activity [22]. Integrating our arguments, we hypothesize:

H1: Strategic flexibility brings in a higher level engagement of bricolage.

### 2.4 The moderating role of environmental turbulence

Environmental turbulence is defined by high levels of uncertainty and unpredictability in consumers' demand and technological change, which means the environment is dynamic and volatile [51]. Technological turbulence and market turbulence are the two primary dimensions of environmental turbulence [24]. The technological turbulence refers to a high rate of change in production technologies and breakthrough product innovation. Market turbulence is characterized by lots of uncertain changes in the composition of customers and rapid demand variation, which means that the new products with different functions will be created to satisfy the consumer's needs in a short product cycle [52]. For example, firms are confronted with the continuously innovative and uncertain environment in the science and technology industry that means firms will constantly change competitive strategies in product creation and manufacturing processes because competitive boundaries are in a state of continuous flux [53]. In a dynamic environment, firms frequently adopt the bricolage to maintain the competitive advantages in the existing marketing and Explore new markets by application of new technologies and new creative products.

We posit further that environmental turbulence strengthens the positive influence of strategic flexibility upon bricolage. When the environment is turbulent, strategic flexibility leads to a higher degree of bricolage. First, the environmental turbulence puts enterprises at risk of losing their resource advantages and existing market segment, and a greater emphasis will be placed on the bricolage activities, such as improvisation and recombining resource at hand, that cuts down on the cost and time of NPD process to meet the consumers' needs rapidly [46]. Accordingly, the environmental turbulence amplifies the importance of resource flexibility—decreasing the cost and time of switching over to another way to use resources for the firm's bricolage activity.

Second, strategic flexibility is considered as one type of dynamic core capability, which makes firms keep a competitive advantage in a turbulent market through building flexibility in the management system, organizational structure, and NPD process. Thus the higher level of environmental turbulence enhances the value creation of strategic flexibility. Strategic flexibility serving as a fundamental principle for coordinating various resources, and functional units enables firms to more frequently engage in improvisation and making do with the low-cost resource at hand and design innovative products without planning or prior preparation. Therefore, we expect that strategic flexibility has stronger relationships with bricolage in the turbulent environment than in a stable one, and so the second hypothesis is:

Hypothesis 2: The environmental turbulence enhances the positive effects of strategic flexibility on bricolage.

### 2.5 Bricolage and product innovation

In the Bricolage framework, entrepreneurs utilize resources at hand to solve problems in new ways with the perception of resource scarcity [15, 48]. Bricolage represents a way of opportunity-formation characterized by organizational capabilities, including improvisation, combinative capabilities, and communication skills. Thereby, bricolage has a substantial effect on the firm's outcomes. For example, bricolage is salient in the bottom-up innovation process as a way of resource mobilization and integration, deploying resources at hand, and coordinating different units [48]. In other words, bricolage is a collective way to initiate innovation pathways by discovering surroundings and related opportunities using whatever is at hand [54]. New product development depends on adequate product knowledge and effective product concept testing, which feedbacks keen insight for new product creation. The bricolage process could use existing networks to create new product ideas and make new materials with handy resources to improve product innovation [38].

The bricolage brings material, ideational, or human inputs to recombine new and old resources, which leads to higher product creativity [46] because the process of recombination and redeploying resources is a primary driver of innovation. Senyard et al. (2014) put forward processes of recombination of existing elements that are fundamental and essential to bring in new products and other innovative outcomes. Coping with new opportunities and challenges, firms attempt to adopt creative combinations of low-cost resources and overcome resource scarcity. Therefore, the enterprises engaging in bricolage will bring about more innovative solutions than those that adopt less bricolage.

Improvisation is another concept relates to bricolage that emerged as a crucial organizational competence [55]. Improvisation capabilities in terms of imagination and creativity lead to new solutions and products, which are appreciated by consumers in the NPD process. For example, the four-phase process model is constructed to illustrate how to conduct effective improvisation and produce creative responses and products in the Tencent Group [56]. Recent studies confirm that improvisation improves the cost efficiency of the enterprise when the teams develop new products by using external and internal market information [57]. Thus, firms engaged in bricolage through improvisation acquire the incremental innovation capability to achieve recombination and reuse of resources for different applications and new purposes [58, 59]. Bricolage serves as a mechanism driving the discovery of product innovation from existing resources [36, 60]. It refers to the use of handy resources, such as social capital, creative ideas, and allocation and integration of resources in new methods [61], and thus bricolage gives rise to a degree of serendipity and results in new product innovation [46].

Besides, bricolage drives corporate entrepreneurship by identifying and exploiting opportunities for entrepreneurial choice and subsequent entrepreneurial activities, like manufacturing resource reconfiguration and new product innovation. Baker and nelson (2005) consider the bricolage as making do by redeploying handy resources to deal with new problems and opportunities [36]. Making do means that there is a bias between action and active participation in coping with challenges and opportunities rather than hesitating to figure out an effective solution by combining handy resources, which suggests that bricolage sometimes enables us to reach remarkable unexpected results in product innovation through active engagement [23]. Therefore, the arguments above lead us to suggest the third hypothesis:

Hypothesis 3: bricolage is positively related to product innovation.

### 2.6 The mediating effect of bricolage

**Fig 1:**
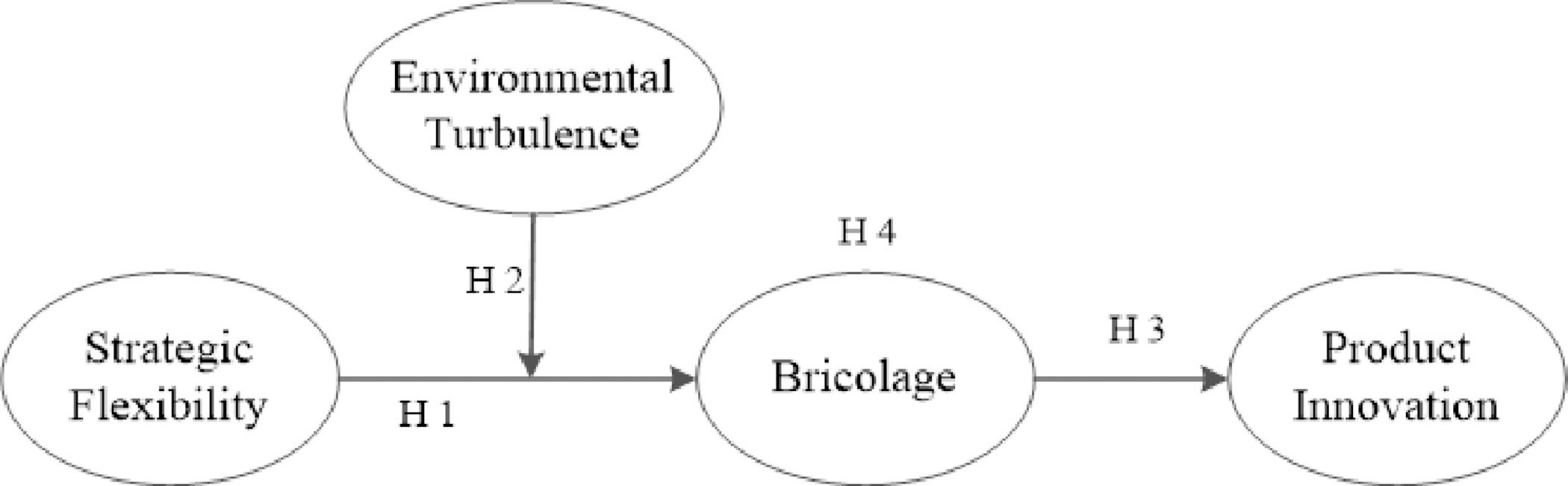
The conceptual model.

Previous studies have drawn a framework to show that strategic flexibility is fundamental to develop new products and create new opportunities for satisfying the rapidly changing demand of consumers [13, 62]. Integrating hypothesis 1 and hypothesis 3, we propose a mediation relationship that bricolage mediates the influence of strategic flexibility on product innovation as depicted in Fig 1. In this research, we regard bricolage as an essential outcome of strategic flexibility. Hence, firms would redeploy existing resources and offer new products in response to changing customer demand via bricolage, [63, 64].

Therefore, given that strategic flexibility affects bricolage, which in turn affects product innovation, we suppose a chain of effect beginning from strategic flexibility to bricolage and finally to product innovation. The majority of existing literatures confirm a direct relation in which strategic flexibility has an impact on product innovation [3, 62]. Recent studies call for investigating the intervening outcomes of strategic flexibility, and thus we reveal the underlying link with the mediating factor bricolage. Thereby, our understanding of how strategic flexibility affects product innovation is strengthened through revealing the underlying channels [62, 20]. It implies that the innovation creation effect of strategic flexibility is path-dependent. Bricolage represents the intermediate channels that result in the ultimate influences of strategic flexibility upon product innovation. Consequently, the corporations have more product strategy options and generate product innovations due to the flexibility in a corporate's chain of the product creation process. Thus, we propose:

Hypothesis 4: Bricolage will mediate the relationship between strategic flexibility and product innovation.

Summarizing all the above hypotheses, we demonstrate the conceptual model in Fig 1 that shows the relationships between strategic flexibility, bricolage, and product innovation. Strategic flexibility as an independent variable is firstly expected to relate to bricolage positively (hypothesis 1), and then the environmental turbulence plays a positive moderating role in the relationship between the strategic flexibility and bricolage (hypothesis 2). It supposes that bricolage, in turn, affects product innovation positively (hypothesis 3), and thus, bricolage will mediate the relationship of strategic flexibility and product innovation (hypothesis 4) because of the direct relationship in which strategic flexibility influences product innovation has been proved in the literature.

## 3. Methods and data

### 3.1 Sampling and data collection

We test the hypotheses by surveying Chinese High-tech industrial firms, and the firms are mainly from electronics, machine manufacturing, information technology, and advanced material. The Chinese economy is in transition with high levels of environmental uncertainty and turbulence, which offers an ideal setting to test the research model. Besides, the government provides many policies to stimulate product innovation, which vary greatly in the east, middle, and west of China. To survive the keen competition, the firm must have a flexible strategy and the resource integration capability to respond to the transitional environment and acquire a sustainable competitive advantage.

Firstly, the questionnaire of the English version is developed based on prior literature. Then, the questionnaire is switched to the Chinese version by two independent translators to ensure conceptual equivalence. The data is collected via on-site interviews from July to December 2017 with the help of a professional research firm. The research firm actually provides consulting services and well-trained interviewers to collect valid and high-quality data in China. Then, we get a directory of the firms from different provinces in east and west China. Secondly, the pre-test with ten firms from Beijing (not included in the final sample) is conducted, and the top managers are asked to answer all the questions and point out the clarity of items. We can get immediate feedback and revise the details of the questionnaire through the face-to-face procedure. And then, the final survey items are improved to ensure the questionnaire sends a clear message to the respondents. The informant is required to recall the corporation's strategic decision process and financial performance in the past three years. The 214 firms in the final sample had an average of 56 employees and five years of history. The respondents both belong to the TMT members, such as CEO, CTO, CMO, and general manager, which suggested they had sufficient experience to offer valid information to the surveys. Eighty percent of respondents had a bachelor's degree, and 70 percent of respondents are 30 years old. There is 70 percent of male respondents.

Common method variance (CMV) would be taken into consideration in the survey data [65, 66]. Different items in the questionnaire are finished by two top managers to reduce the potential CMV and ensure that the managers are familiar with the firm's operation and strategic making process. We gain different information from different resources. Specifically, the respondents provide information about the latent variable, such as strategic flexibility, bricolage, and environmental turbulence. Information about firm age, size, industry, and revenue is from the archival data provided by the firm. The different data sources will effectively soften the common method bias. It is a common method to adopt the Harman one-factor test to indicate the level of CMV [67], and so we conduct the test and extract the first factor, which explains 31.18% of the variance. Hence, CMV is at a reasonable level. Furthermore, our hypotheses involving interactions, if supported, offer further evidence that CMV was not a problem because respondents are not likely to use an "interaction-based theory" to systematically bias their answers to produce our results [68].

There are 300 firms in the survey sample, and 86 firms do not finish their surveys due to dealing with emergencies or being not known to answer all the items. Thereby, we successfully gain 214 firms in the final sample, which has an effective response rate of 71.33% after dropping cases with missing data. The sample main contains SMEs, whose total number of employees is less than 500, and the average firm age is less than 4.5 years. The largest industry segment is an information technology (74.30%), followed by material technology (8.4%), biological medicine (8.9%), and machine manufacturing (8.4%).

### 3.2 Measures

We adapted construct scale from previous literature and changed the word and sentence to improve the understanding of Chinese informants [6, 17, 24, 36, 69]. We apply the seven-point Likert-type scale to measure these items with endpoints of "strongly disagree" and "strongly agree." The construct scales are evaluated, ranging from 1 = " strongly disagree" to 7 = " strongly agree." The respondents are asked to pay attention to the degree to which they agreed with different items depending on their firms' performance compared with their competitors over the past three years.

We measure product innovation by utilizing five items to following the previous literature [7, 69]. The respondents are required to answer the questions and evaluate the extent to which their firms are successful compared to their major competitors in terms of (1) quickly launching new products into the market, (2) applying advanced production facilities, (3) having a better market response in product modifications and innovations, (4) having state-of-the-art technology in products, (5) being more successful with their product innovations.

To measure strategic flexibility, we use the survey items developed by Dai and Goodale (2018) based on Sanchez's theoretical work [17]. Strategic flexibility is defined as flexibility in resource use and coordination, responding to the dynamic environments [26]. Resource flexibility and coordination flexibility are the two dimensions of strategic flexibility [11]. Resource flexibility focuses on identifying and obtaining different uses of flexible resources, which brings about various strategic options for enterprise managers. So we apply the range of resource use and the difficulty, time, and cost needed in switching the different use of resources. Coordination flexibility is attributed to developing flexibility in coordinating the use of resources, and so we adopt the "reconfiguring chain of resource and deploying resources via organizational structures" to measure it [6].

We adapt Baker and Nelson's (2005) concept of bricolage and develop four items referring to Senyard's research [25] to measure the bricolage. The items are "dealing with new challenges by applying a combination of our existing resources, applying combinations of resources at hand for new operations, putting together workable solutions from our existing resources, and using any existing resources that seem useful to respond to a new problem or opportunity. It captures most elements of the bricolage definition-"making do by applying combinations of resources at hand to new problems and opportunities."

Environmental turbulence is measured by identifying the frequency and speed of technological change and evaluating changes in customer's preferences [24, 70]. The technological turbulence focuses on the change rate of technology in the industry and the substantial opportunities in the technological changes [71]. The market turbulence shows that it changes quickly in customers' product preferences over time, and the firm is confronted with demand variation [72].

To explain the influences of extraneous variables, we regard the sales, size, firm's age, firm's industry, product stage, and business strategy as control variables. The number of employees is defined as the indicator of firm size. Firms with fewer than ten employees are given a score of 1. The firms with 11–50 employees are given 2, 51–100 employees are given a score 3, 101–500 employees are given a score 4, and firms with over 500 employees are score 5. The firm's age is calculated by the number of years the firm has been founded. The product stage refers to the research of Chang (2019) that the development stage is given the score 1, and the mature stage is given the score 0 [73]. For the business strategy, the dummy variable is applied to represent the prospector strategy and defender strategy using the defender strategy as the baseline. Similarly, we use 3 dummy variables to handle four industries.

## 4. Results

### 4.1 Descriptive statistics

The descriptive statistics and a correlation matrix demonstrate the key variables and their inter-relationships shown in Table 1. The correlation between explanatory variables is less than the rule-of-thumb cutoff 0.7, and the maximum variance inflation factor (VIF) in the full model is 1.77, which is less than the critical value of 10, minimizing multicollinearity. Strategic flexibility relates positively to bricolage (β = 0.608, p<0.01), offering initial evidence to Hypothesis 1.

**Table 1:** Descriptive statistics and correlation matrix.

Variables	Mean	SD	1	2	3	4	5	6	7	8
1.Firmage	4.561	2.008								
2.Sales	15.72	1.374	0.112							
3.Firm Size	2.397	0.928	-0.009	0.510***						
4.Stage	3.988	1.421	-0.029	0.197***	-0.094					
5. Product innovation	5.147	1.038	0.068	0.229***	0.258***	-0.283**	**0.79**			
6.Bricolage	5.661	0.772	-0.036	0.138**	0.133*	-0.034	0.554***	**0.7**		
7. Strategic Flexibility	5.388	0.764	0.037	0.019	0.044	-0.248*	0.497**	0.608**	**0.71**	
8.Environmental Turbulence	5.668	0.783	0.014	0.111	0.087	-0.059	0.436**	0.466**	0.523**	**0.71**

N = 214;

* p< 0.01,

**p<0.05

*** p< 0.01.

The data on the diagonal in bold font is the square root of the average variance extracted of the construct.

### 4.2 Reality and validity

Firstly, the exploratory factor analysis is conducted for each multi-item scale by applying the principal component method with varimax rotation, and then we make to extract four components [74] including strategic flexibility, bricolage, product innovation, and environmental turbulence. The reliability of all reflective constructs is examined through using indicators of Cronbach's alpha and composite reliabilities (CR). All constructs have high reliability, and the alpha values are over 0.7 in Table 2. Secondly, the confirmatory factor analysis is estimated to examine the validity of reflective constructs and assess our model fit. After dropping the items of low factor loadings, our model achieves a satisfactory fit to the data (Chi-square/df = 1.44, goodness-of-fit index [GFI] = 0.93, comparative fit index [CFI] = 0.96, incremental fit index [IFI] = 0.92; root mean square error of approximation [RMSEA] = 0.045). The value of items factor loading and AVEs are above or close to 0.5, which shows a high convergent validity. The discriminant validity is examined by comparing the square root of the AVE with the correlations. As shown in Table 1, none of the correlations are higher than the square root of AVE for each construct, indicating the model has good discriminant validity.

**Table 2:** Measurement reliability and validity.

Variables	Loading	Cronbach's α	Composite Reliability	AVE
**Strategic Flexibility (SF)**		0.772	0.84	0.51
There is a larger range of alternative uses to which a resource can be applied.	0.68			
The costs and difficulty of switching from one use of a resource to an alternative use.	0.82			
The time required to switch to alternative resource use is low.	0.78			
Identify environmental changes and reconfiguring chains of resources the firm can use in developing, manufacturing, and delivering its intended products to targeted markets	0.62			
Deploy resources through organizational structures that support the firm's product strategies.	0.64			
**Bricolage**		0.78	0.79	0.49
We deal with new challenges by applying a combination of our existing resources and other resources inexpensively available to us.	0.73			
Applying combinations of resources at hand for new operations	0.65			
When we face new challenges, we put together workable solutions from our existing resource	0.69			
We use any existing resource that seems useful to respond to a new problem or opportunity.	0.72			
**Product innovation (PI)**		0.89	0.90	0.63
Quickly launching new products into the market	0.77			
Our production facilities are more advanced than those of our competitors	0.85			
Compared with our competitors, our product modifications and innovations have a better market response	0.81			
Our products are of state-of-the-art technology.	0.77			
Compared with our competitors, we have more success with their product innovations.	0.78			
**Environmental Turbulence (ET)**		0.72	0.80	0.50
The technology in this industry is changing rapidly	0.64			
Technological changes provide substantial opportunities in this industry	0.82			
In our kind of business, customers' product preferences change quite a bit over time.	0.67			
We are witnessing demand for our products and services from customers who never bought them before	0.69			

### 4.3 Regression analysis

The hierarchical regression model is used to test our hypotheses, and the empirical results are shown in Table 3. Before creating interaction terms, the independent and moderator variables are centralized in order to decline potential multicollinearity [75, 76]. The variance inflation factor (range = 1.03–1.39) is examined that well below the cutoff of 10, which suggests that there is No serious multicollinearity. The basic models are model 5 and model 6, including the bricolage, product innovation, and control variables.

**Table 3:** Regression analysis results.

	1	2	3	4	5	6
	bricolage	bricolage	innovation	innovation	bricolage	innovation
Firm age	-0.031 (-1.30)	-0.042* (-1.78)	0.034 (1.23)	0.025 (0.87)	-0.022 (-0.70)	0.018 (0.51)
Sales	0.003 (0.07)	0.011 (0.29)	0.075 (1.28)	0.086 (1.49)	-0.025 (-0.47)	0.057 (0.80)
Firm Size	0.079 (1.64)	0.065 (1.36)	0.104 (1.41)	0.107 (1.47)	0.116* (1.74)	0.188** (2.08)
Strategy	0.075 (0.69)	0.050 (0.48)	0.138 (0.75)	0.081 (0.46)	0.276* (1.96)	0.338* (1.80)
Product Stage	-0.079 (-0.77)	-0.100 (-0.97)	-0.209* (-1.88)	-0.186* (-1.74)	-0.190 (-1.39)	-0.346** (-2.41)
Strategic Flexibility	0.596*** (9.61)	0.499*** (7.26)		0.341*** (2.63)		0.647*** (6.44)
Environmental Turbulence		0.208*** (3.09)				
SF×ET		0.172** (2.11)				
Bricolage			0.723*** (8.04)	0.512*** (4.09)		
VIF	1.301	1.380	1.300	1.270	1.300	
Adjust R^2^	0.393	0.431	0.341	0.390	0.045	0.126
△R^2^	0.348	0.386	0.215	0.264		
N	214	214	214	214	214	214

*p < 0.1,

**p < 0.05,

***p < 0.01.

Hypothesis 1 predicts that strategic flexibility is positively associated with bricolage. The analysis results are reported in Table 3. It shows that strategic flexibility has a positive and significant effect (b = 0.596, p<0.01) on the bricolage in model 1. Therefore, the result fully supports Hypothesis 1.

**Fig 2:**
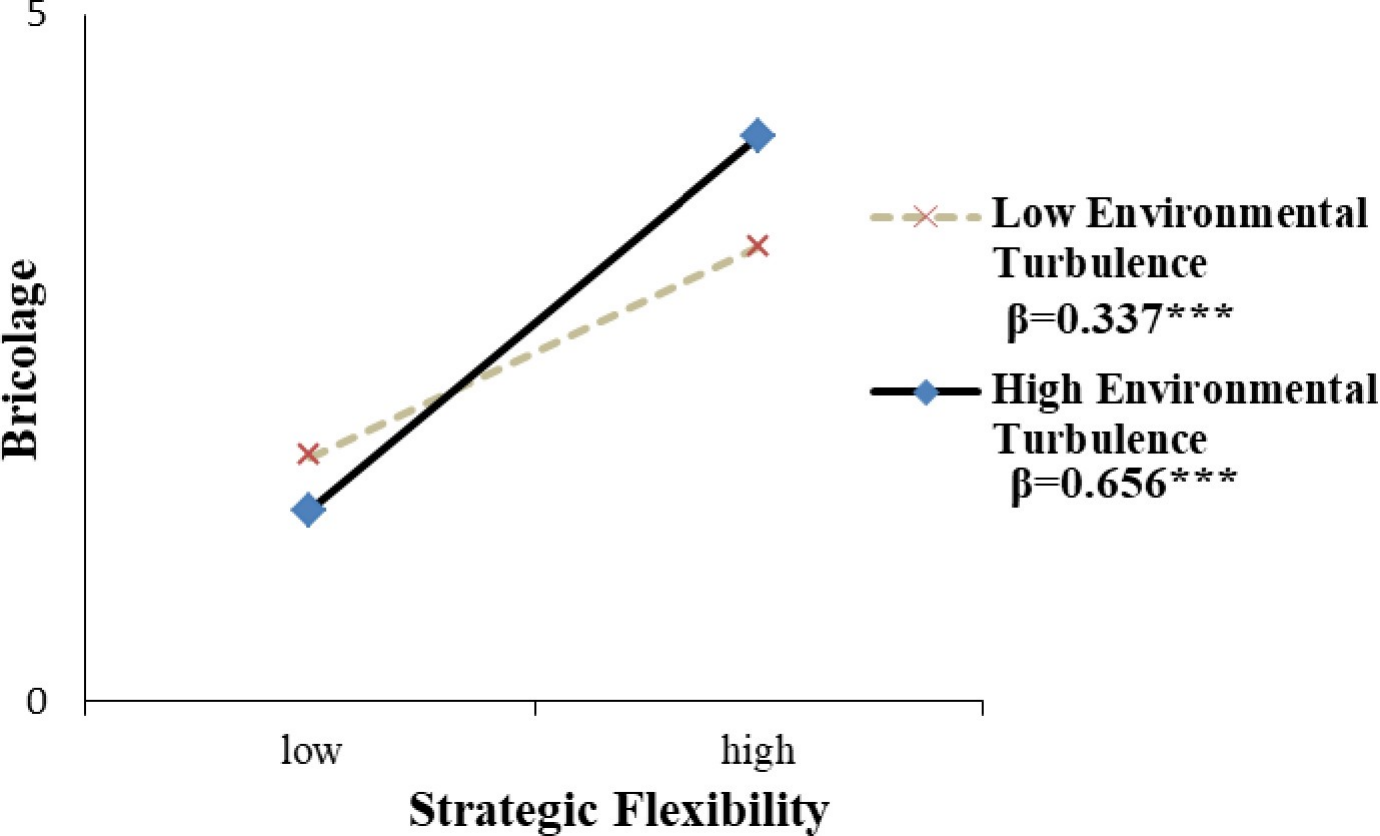
The moderating effect plots.

In Hypothesis 2, we expect that environmental turbulence enhances the positive effects of strategic flexibility on bricolage. The result suggests that the moderating effect of environmental turbulence is positive and significant (b = 0.172, p<0.05) in model 2. We refer to the research procedure of Ailen and West (1991) to acquire more insights into the interaction effects of hypothesis 2. As reported in Fig 2, the simple slope test is conducted with the line graph describing the relationship. In the test, the environmental turbulence is split into two groups-low (one standard deviation below the mean) and high (one standard deviation above the mean), and then we evaluate the effect of strategic flexibility on bricolage on both levels. As reported in Fig 2, the bricolage increases more quickly when the degree of environmental turbulence shifts from low to high. This implies that environmental turbulence plays a positive moderating role in the relationship between strategic flexibility and bricolage. Thus, hypothesis 2 is supported.

In hypothesis 3, we examine the impact of bricolage upon product innovation. Like the model 3 shows, bricolage relates to product innovation positively (b = 0.723, p<0.01), which is in support of hypothesis 3.

Hypothesis 4 predicts a mediated relationship between strategic flexibility and product innovation. According to Baron and Kenny's test, results for the first condition (predictor-mediator) that demonstrates that strategic flexibility relates to bricolage significantly in hypothesis 1. Result for the second condition (mediator-outcome, see hypothesis 3) shows that bricolage has a positive impact on product innovation. Finally, the third condition (predictor-mediator-outcome) shows that strategic flexibility has a positive influence on product innovation significantly (b = 0.238, p<0.1) when bricolage is included in the equation in model 4. This pattern of result indicates partial mediation of bricolage, thus supporting hypothesis 4. What is more, we adopt the Sole-Goodman mediation tests, the indirect effects of the test (b = 0.330, p<0.01) provide significant evidence of mediation effect, with the bootstrapped 95% of CIs not containing zero (percentile CI = 0.166, 0.05). Bricolage partially mediates approximately 49% of the total effect of strategic flexibility on product innovation, which supports hypotheses 4.

## 5. Discussion

Employing the process of strategic flexibility helps the firm benefit from the product innovation and sustain the competitive advantage [6, 7, 22, 26]. This paper builds upon the strategic flexibility literature from a unique perspective by investigating how a firm's entrepreneurial activity-bricolage mediates the strategic flexibility's influence on product innovation. Based on the RBV, the results illustrate that strategic flexibility has a positive impact on bricolage, and the environmental turbulence enhances the positive effect using the survey data in China. Furthermore, our finds confirm that bricolage affects product innovation positively and mediates the relationship between strategic flexibility and product innovation. What is more, it reveals the antecedent of bricolage, which regarded as a path to innovativeness for enterprises under resource constraints in Desa and Basu's (2013) study. These findings provide a significant extension to the work of Li et al. (2010) on the relationship between strategic flexibility and product innovation. Below, the theoretical and practical implications are discussed based on our finds.

Our research advances knowledge about the strategic flexibility and product innovation in an uncertain environment and makes four essential contributions. The first key contribution lies in connecting theory on strategic flexibility with existing product innovation literature. Although evidence emphasizes the positive role of flexibility in product innovation and financial performance [6, 62, 7], most of the work on strategic flexibility has focused on the indirect effect or moderating effect on the innovativeness as one type of dynamic capability. In contrast, the direct effect on product innovation has not received much attention. Our findings reveal one pathway via which strategic flexibility influence product innovation. The effect of strategic flexibility on product innovation is a complicated pattern, and demystifying it needs the intermediate factors that are routes for the path-dependent resource value creation role of strategic flexibility. Extant literature exclusively on the ultimate output performance, such as product innovation [6, 7] and financial performance [1], competitive advantage [9, 10], not explicitly highlight the intermediate factors. We introduce the bricolage as the intervening channels to expand this stream of studies. These findings provide a broader potential value for strategic flexibility in organizational behavior and performance. Furthermore, bricolage contributes to a better understanding of why and how strategic flexibility may lead to product innovation. So it deserves extra attention to foster and develops strategic flexibility in the turbulence environment. We contribute the product innovation literature by confirming a "capability-behavior-innovation" mechanism, which corroborates evidence suggesting that the firms with the dynamic capability and entrepreneurial orientation have a higher level of product innovation [6, 77].

Second, this research advances our understanding of boundary conditions of strategic flexibility's effect and the environment under which the benefits of strategic flexibility come into play. While the impacts of strategic flexibility have been extensively confirmed [2, 18, 78], agreement on the impact of strategic flexibility is still ambiguous. Strategic flexibility at the cost of efficiency needs an organizational structure that is not easily created [35]. We find that environmental turbulence plays a positive, moderating role in the relationship between strategic flexibility and bricolage. While dynamic and turbulent environment provides more opportunities and external knowledge to reconfigure and recombine existing flexible resource for improvisation. This study elucidates that strategic flexibility helps the firm gain benefit from the turbulent and dynamic environment. The uncertain environment provides the fertile ground for the competitive advantage brought by the bricolage.

Third, we extend the bricolage literature by investigating strategic flexibility as the antecedent of bricolage and provide new evidence regarding the bricolage as a path to innovativeness for resource-constrained firms [25]. Previous studies have mainly focused on the influences of service entrepreneurship [49], exploratory orientation [41], and opportunity recognition [79] on the bricolage. Interestingly, the assumption of bricolage is the condition of resource scarcity [15], and combining existing resources for new opportunity unlocks a form of new valuation creation. However, scarcity is a necessary, but not sufficient condition. We interpret the strategic flexibility as the prerequisite of bricolage, which complements the insufficient assumption [15]. Namely, strategic flexibility plays an essential role in the entrepreneurial activity to combine existing resources for a new purposed.

The critical managerial implications are also highlighted based on our findings. Strategic flexibility affects bricolage and product innovation positively. In that regard, our study helps enterprises managers gain a broader understanding of flexible resource and organizational structure in changing circumstances. For example, strategic flexibility is crucial for entrepreneurial behavior and fostering entrepreneurs' efficacy in developing new products. Managers should develop flexibility in resource deployment and coordination, which leads to making do by applying a combination of handy resources to new opportunities and, in turn, brings about more innovation in new product development. The ZTE Corporation, the leader in the Chinese telecommunication industry, lacked alternative resources and shut down when partial suppliers stop providing chips because of the uncertain environment in the 2018 trade war. In contrast, the Huawei Company adopted the flexible strategy and developed the alternative chips and operating system, which led to the new creative, smartphone, and avoided a shutdown. To acquire higher flexibility, firms should design flexible organizational structures and develop the ability to utilize external knowledge [17].

Finally, the findings confirm that environmental turbulence interacts with flexibility in a way that facilitates bricolage. Actually, the environmental turbulence incurs both resource scarcity and new opportunity [15]. It implies that managers should creatively tackle the dynamic environment that brings in the external knowledge and various market opportunities. Indeed, strategic flexibility help firms gain benefits from environmental turbulence in term of demand variation and technical change [18].

This paper has some limitations that provide opportunities for further research. First, the cross-sectional data in the research perhaps limit the investigation of the causal inferences of strategic flexibility and bricolage. Although existing theories and empirical results support our hypotheses, causality cannot be ascertained with a correlational study. The longitudinal research using archival data would improve the robustness of our finds. Second, our sample only focuses on Chinese corporations, and our results do not apply in other emerging economies. Nevertheless, it remains an open question whether our findings can be generalized to other emerging economies where the environment is uncertain and dynamic with rapid economic growth. Then China has a collectivist culture, which influences the manager's attitude toward uncertainty and environmental turbulence. So it suggests the need for future work to make a comparison between the firms in an individualistic culture and collectivist culture. Third, we refer to the construct of strategic flexibility developed by Sanchez (1995) [26] and regard the strategic flexibility as the capability to maneuver available resources easily. However, there exits another application of strategic flexibility used as a scenario analysis tool. It emphasizes that organizational leaders and managers require flexibility to adjust decisions within given constraints according to the view of decision-making process [8]. So we also encourage future studies to investigate other outcomes of strategic flexibility, such as decision-making and planning.

In conclusion, environmental turbulence makes strategic flexibility a particularly significant capability for product competition [7, 26]. However, scholars have not unpacked the mediating mechanism on the flexibility-innovation relationship from the view of the resource utilization process. We identified a critical intermediator-bricolage that channels the influence of strategic flexibility on innovation. In doing so, we hope the finds will inspire and serve as a springboard for future studies on the underlying channels that delineate the flexibility-innovation relationship.

## Supporting information

S1 File Data and stata program. (RAR)
